# LIFECOURSE STRESS EXPOSURES, RESILIENCE, AND BIOBEHAVIORAL MECHANISMS UNDERLYING DISPARITIES IN COGNITIVE AGING

**DOI:** 10.1093/geroni/igae098.0621

**Published:** 2024-12-31

**Authors:** Ruijia Chen

**Affiliations:** Boston University, Lexington, Massachusetts, United States

## Abstract

Stress exposures across the lifespan, such as childhood adversities and financial strain, may contribute to social disparities in cognitive outcomes. Individuals from socially disadvantaged backgrounds tend to be exposed to more stressors than their advantaged counterparts. Prior research has indicated that psychosocial stressors are key determinants of physical and mental health as well as health disparities. However, their roles in Alzheimer’s Disease and Related Dementias (ADRD) risk have received considerably less attention. In addition to understanding and intervening directly on stressors, elucidating modifiable factors, such as social networks, further along these pathways may offer additional intervention avenues. Greater social networks have been consistently noted as predictors of lower ADRD risk, yet the structure and impact of social networks may vary across racial and ethnic groups, making it essential to assess the associations between social networks and ADRD risk in diverse racial and ethnic groups. Psychosocial stressors may lead to sleep disturbances (e.g., sleep apnea and insomnia), biobehavioral factors that disproportionately affect racial and ethnic minorities. With the availability of effective treatments for some types of sleep disturbances, evaluating how these disturbances contribute to ADRD risk across different racial and ethnic groups could identify novel targets for reducing disparities in ADRD. This presentation will cover a series of studies applying advanced epidemiologic methods, including causal inference, to investigate the roles of life course stress and resilience factors in contributing to disparities in cognitive aging.

